# Linking Joint Impairment and Gait Biomechanics in Patients with Juvenile Idiopathic Arthritis

**DOI:** 10.1007/s10439-019-02287-0

**Published:** 2019-05-20

**Authors:** Erica Montefiori, Luca Modenese, Roberto Di Marco, Silvia Magni-Manzoni, Clara Malattia, Maurizio Petrarca, Anna Ronchetti, Laura Tanturri de Horatio, Pieter van Dijkhuizen, Anqi Wang, Stefan Wesarg, Marco Viceconti, Claudia Mazzà, for the MD-PAEDIGREE Consortium

**Affiliations:** 1 Department of Mechanical Engineering, University of Sheffield, Sheffield, UK; 2 INSIGNEO Institute for In Silico Medicine, University of Sheffield, Sheffield, UK; 3 Department of Civil and Environmental Engineering, Imperial College London, London, UK; 4 Department of Mechanical and Aerospace Engineering, “Sapienza” University of Rome, Rome, Italy; 5 Pediatric Rheumatology Unit, IRCCS “Bambino Gesù” Children’s Hospital, Passoscuro, Rome, Italy; 6 Pediatria II - Reumatologia, Istituto Giannina Gaslini, Genoa, Italy; 7 Movement Analysis and Robotics Laboratory (MARLab), Neurorehabilitation Units, IRCCS “Bambino Gesù” Children’s Hospital, Passoscuro, Rome, Italy; 8 UOC Medicina Fisica e Riabilitazione, IRCCS Istituto Giannina Gaslini, Genoa, Italy; 9 Department of Imaging, IRCCS “Bambino Gesù” Children’s Hospital, Passoscuro, Rome, Italy; 10 Paediatric Immunology, University Medical Centre Utrecht Wilhelmina Children’s Hospital, Utrecht, The Netherlands; 11 Visual Healthcare Technologies, Fraunhofer IGD, Darmstadt, Germany; 12 Department of Industrial Engineering, Alma Mater Studiorum - University of Bologna, Bologna, Italy; 13 Laboratorio di Tecnologia Medica, IRCCS Istituto Ortopedico Rizzoli, Bologna, Italy

**Keywords:** Biomechanics, Musculoskeletal, Gait analysis, MRI, Musculoskeletal modelling, Lower-limb, Juvenile arthritis, Opensim

## Abstract

Juvenile Idiopathic Arthritis (JIA) is a paediatric musculoskeletal disease of unknown aetiology, leading to walking alterations when the lower-limb joints are involved. Diagnosis of JIA is mostly clinical. Imaging can quantify impairments associated to inflammation and joint damage. However, treatment planning could be better supported using dynamic information, such as joint contact forces (JCFs). To this purpose, we used a musculoskeletal model to predict JCFs and investigate how JCFs varied as a result of joint impairment in eighteen children with JIA. Gait analysis data and magnetic resonance images (MRI) were used to develop patient-specific lower-limb musculoskeletal models, which were evaluated for operator-dependent variability (< 3.6°, 0.05 N kg^−1^ and 0.5 BW for joint angles, moments, and JCFs, respectively). Gait alterations and JCF patterns showed high between-subjects variability reflecting the pathology heterogeneity in the cohort. Higher joint impairment, assessed with MRI-based evaluation, was weakly associated to overall joint overloading. A stronger correlation was observed between impairment of one limb and overload of the contralateral limb, suggesting risky compensatory strategies being adopted, especially at the knee level. This suggests that knee overloading during gait might be a good predictor of disease progression and gait biomechanics should be used to inform treatment planning.

## Introduction

Juvenile Idiopathic Arthritis (JIA) is a group of paediatric chronic diseases of unknown aetiology, particularly affecting the knee and ankle joints,^28^ which can lead to cartilage damage due to inflammation, articular malposition and altered mobility.^19^,^28^ Current practice to quantify disease activity in JIA is based on composite tools such as the Juvenile Arthritis Disease Activity Score (JADAS^7^). The JADAS consists of the following items: the total number of joints with active arthritis, the physician and the patient’s/parent’s global assessment of the disease and the erythrocyte sedimentation rate as an inflammatory marker. The physician and patient’s/parent’s global assessment constitute a subjective element of evaluation of joints status and mobility, and as such can present strong disagreement.^24^,^29^

Medical imaging has been proposed as an alternative in improving the assessment of JIA with respect to traditional clinical examination with ultrasound techniques being used to quantify the tendon and joint synovial inflammation, or cartilage and bone integrity.^6^ More recently, Magnetic Resonance Imaging (MRI) has been introduced to support early diagnosis of JIA thanks to more reliable quantification of synovitis, bone marrow oedema, and bone erosions.^17^,^26^ Image-based techniques, however, can only provide information about local impairment, as usually assessed in unloaded static conditions, and as such are not necessarily informative in terms of consequent functional alterations that could explain different patterns of pathology progression. For this reason, gait analysis techniques have been suggested as a tool to functionally characterise alterations in the joint kinematics and kinetics of patients with JIA.^3^,^13^,^15^,^22^ These studies reported hyper-flexion of the hip and knee joints and reduced plantarflexion of the ankle joint, with associated reduction in ankle moment and power as common gait pattern traits of in JIA. These alterations returned to normal after treatment in the less severe cases, suggesting a clear connection between JIA activity and functional impairment.^3^,^13^,^15^ Unfortunately, no insight into the specific causes of the observed biomechanical alterations that could have explained the absence of a response to treatment in more severe cases was provided. Since internal joint loading is directly related to bone and cartilage loading, it can be hypothesised that its estimate can provide further insight on the link between joint inflammation and impaired walking function. Understanding this link would support more accurate diagnosis and specific treatment planning. Musculoskeletal (MSK) modelling of the lower limb can be used for this purpose.^42^

Several MSK modelling approaches have been proposed in the literature for representing individual patients, from the scaling of generic models to match the subject’s anatomical features^1^ to more detailed image-based techniques.^2^,^16^,^40^ The latter has proved to be a feasible approach for the investigation of lower limb biomechanics in juvenile populations^20^,^22^ and can provide tools to gain insight in disease mechanisms, especially when MSK dysfunction appears causing functional limitations and altered locomotion.^8^,^27^,^31^,^32^

The aim of this paper is to provide further insight into the relationship of disease activity and joint impairment to altered joint loading in children with JIA, and to highlight compensatory strategies that potentially lead to joint damage. To this purpose, we will first establish the repeatability and reproducibility of a patient specific MSK modelling approach previously proposed for the analysis of juvenile gait.^20^ This approach will then be used to investigate the relationship between joint involvement (intended as presence of inflammation and/or cartilage damage in one or more of the lower limb joints) and the hip, knee and ankle joint contact forces (JCFs) in a group of children with JIA. We hypothesised that in the presence of an active disease, where inflamed joints need to be protected to prevent pain, a reduction of the internal loads should be observed. The adopted protection strategies, however, might also lead to a compensation causing overloading of other joints in the same or opposite limb.

## Materials and Methods

### Subjects and Data Acquisition

Eighteen participants (5 males, 13 females, age: 12 ± 3 years, mass: 50.2 ± 17.3 kg, height: 150 ± 16 cm, Table 1) diagnosed with JIA were recruited from two different children’s hospitals (“Bambino Gesù” Children’s Hospital, Rome, Italy, and Istituto Giannina Gaslini, Genoa, Italy). The inclusion criteria were ankle arthritis in new onset JIA or ankle involvement in long lasting JIA (as assessed by clinical observation) and age between five and sixteen years. The ethical committees of both hospitals approved the study and written informed consent was obtained by the patients’ carers.

**Table 1:** Patients’ anthropometric and clinical details.

Patient	Gender (F/M)	Age (year)	Height (m)	Weight (Kg)	Sub-type	MRI_Index_	
						Right	Left
1	F	10	1.39	41	PsA	0	3
2	F	15.5	1.61	68	Ext oligo	3	3
3	M	14	1.74	76.5	Poly-	0	0
4	F	11	1.45	54	Oligo	0	1
5	F	18.5	1.59	68	Ext oligo	3	0
6	F	16.5	1.68	83	Ext oligo	2	5
7	F	14.5	1.65	54.5	PsA	3	5
8	F	11	1.31	26.6	Poly-	2	0
9	F	14	1.63	63.8	Poly-	0	0
10	F	9	1.29	32.5	Poly-	2	1
11	M	10	1.5	37	Oligo	1	2
12	F	7	1.28	23	UndA	2	1
13	M	7.5	1.17	35.7	Oligo	1	1
14	F	13	1.68	49	Oligo	0	2
15	M	12.5	1.55	45.6	Oligo	0	0
16	M	10	1.36	32	Oligo	1	3
17	F	13.5	1.56	54.5	Oligo	0	0
18	F	13.5	1.54	63.5	Poly-	0	0
Average	–	11.9	1.48	47.8	–	–	–
SD	–	3.2	0.17	18.6	–	–	–
Total	15F	–	–	–	–	–	–

*Oligo* persistent oligoarticular JIA, *Ext oligo* extended oligoarticular JIA, *PsA* psoriatic arthritis, *Poly-* rheumatoid-factor-negative polyarticular JIA, *UndA* undifferentiated arthritis

**Figure 1:**
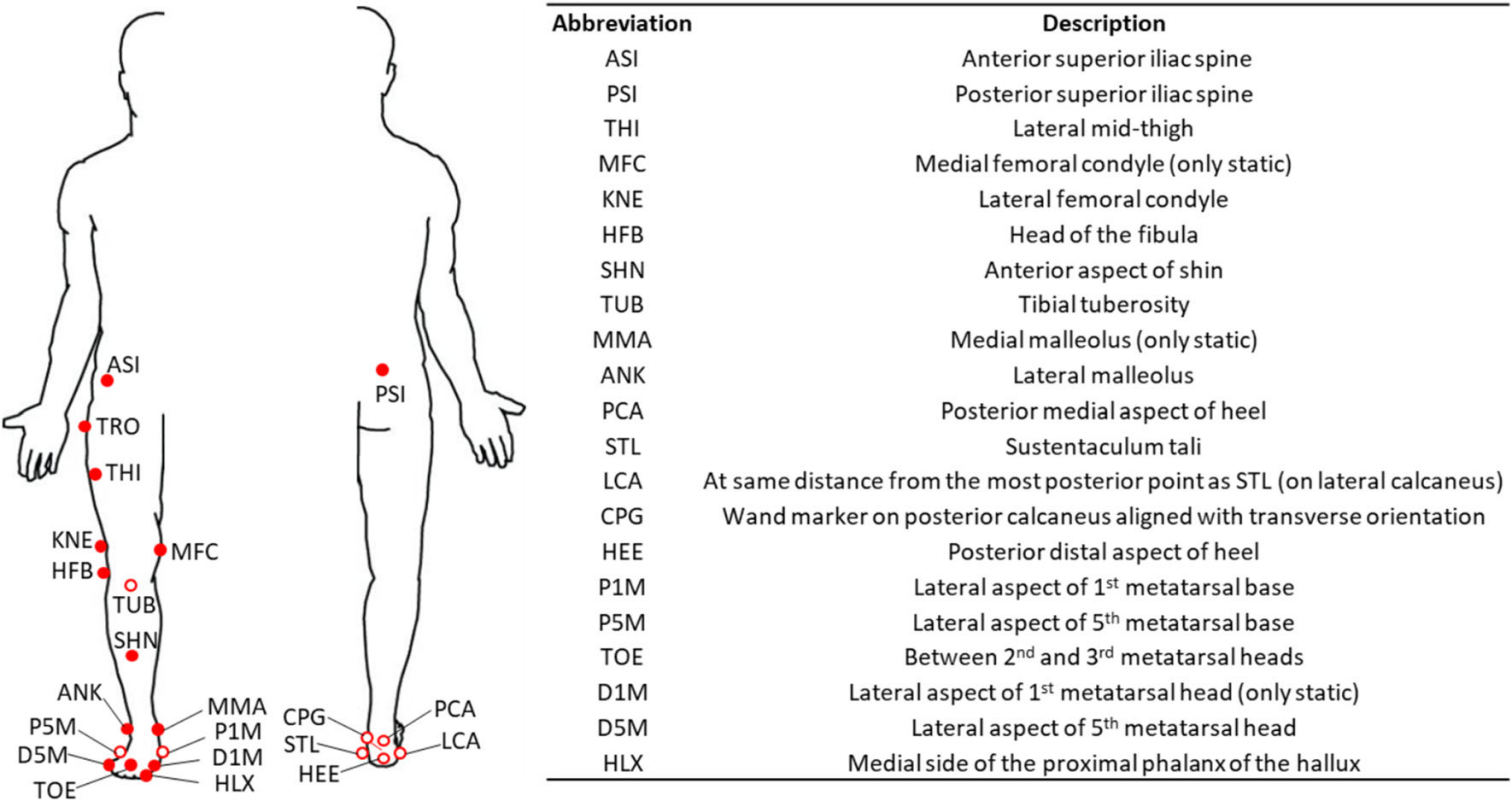
Experimental markers used in the stereophotogrammetric protocol (filled and empty dots) and retained during the imaging (filled dots) and relevant description.

Gait analysis data were collected in the two different hospitals using movement analysis based on infrared optical stereophotogrammetry. An 8-camera system (MX, Vicon Motion System Ltd, UK, 200 Hz) with two force platforms (OR6-6, AMTI, USA, 1000 Hz) was used in Rome and a 6-camera system (Smart DX, BTS Bioengineering, Italy, 100 Hz) with two force platforms (Kistler, UK, 1000 Hz) was used in Genoa. The marker-set was a combination of the Vicon PlugIn gait (Vicon Motion System) and the modified Oxford Foot Model (mOFM) protocols,^35^ with a total of fourty-four markers (Fig. 1). A subset of twenty-eight markers was retained during a following MRI exam (see Modenese *et al*.^20^ for detailed protocol) including a full lower limb 3D T1-weighted fat-suppression sequence (e-THRIVE) with 1 mm in-plane resolution and 1 mm slice thickness.

### Musculoskeletal Modelling Procedure

Eighteen lower limb patient-specific MSK models were built following the procedure described in Modenese *et al*.^20^ Bone geometries were segmented from the MRI with a statistical shape modelling approach.^37^ The anatomical models were built by one expert operator using specialised software (NMSBuilder^39^). Nine body segments were included in the model, namely one pelvis and bilateral femur, tibia, talus and foot segments. The inertial properties of each segment were computed accounting for both bone and soft tissue densities.^41^ Eight joints were modelled as ideal ball-and-socket (hip) joint, or ideal hinge (knee, ankle, subtalar). The articular surfaces were identified and isolated in Meshlab^5^ and the joints’ axes of rotation were defined with a morphological fitting approach using a least square difference minimisation in MATLAB (v9.1, R2016b, The MathWorks Inc., USAMathWorks, USA) and following the ISB conventions.^43^ Muscle attachments and *via* points were calculated through a supervised atlas registration approach^40^ based on a reference model^11^ and manually adjusted against the MRI if needed.

Musculotendon parameters were modelled as Hill-type muscle elements.^38^ Optimal fibre length (*l*_opt_), tendon slack length (*l*_tendon_) were scaled to maintain the *l*_opt_/*l*_tendon_ ratio as in the “gait2392” generic model^11^ available with the OpenSim distribution. Pennation angle was set according to the value in the “gait2392”^11^ and maximal contraction velocity was set to 10 fibres per second.^38^ Maximal isometric force (*F*_max_) was linearly scaled based on the ratio between the lower-limb mass of the subject (derived from the MRI) and of the generic model.^11^ The force–length–velocity (FLV) relationship was not considered during the simulations, thus neglecting contraction dynamics.^20^

The experimental markers visible in the MRI were included into the model as virtual markers and then registered to those used for the gait analysis within OpenSim,^10^ where gait was simulated using the Inverse Kinematics and Inverse Dynamics routines. Gait data were normalised over a gait cycle, identified from subsequent heel strikes of the same limb, which were determined either from the force platform or from the foot markers. Joint powers (JPs) were calculated as the product of joint moment and angular velocity. The Static Optimisation tool was used to estimate muscle activations and forces and the Joint Reaction Analysis tool^36^ was then used to estimate the JCFs (intended as the norm of the reaction force vector).

**Figure 2:**
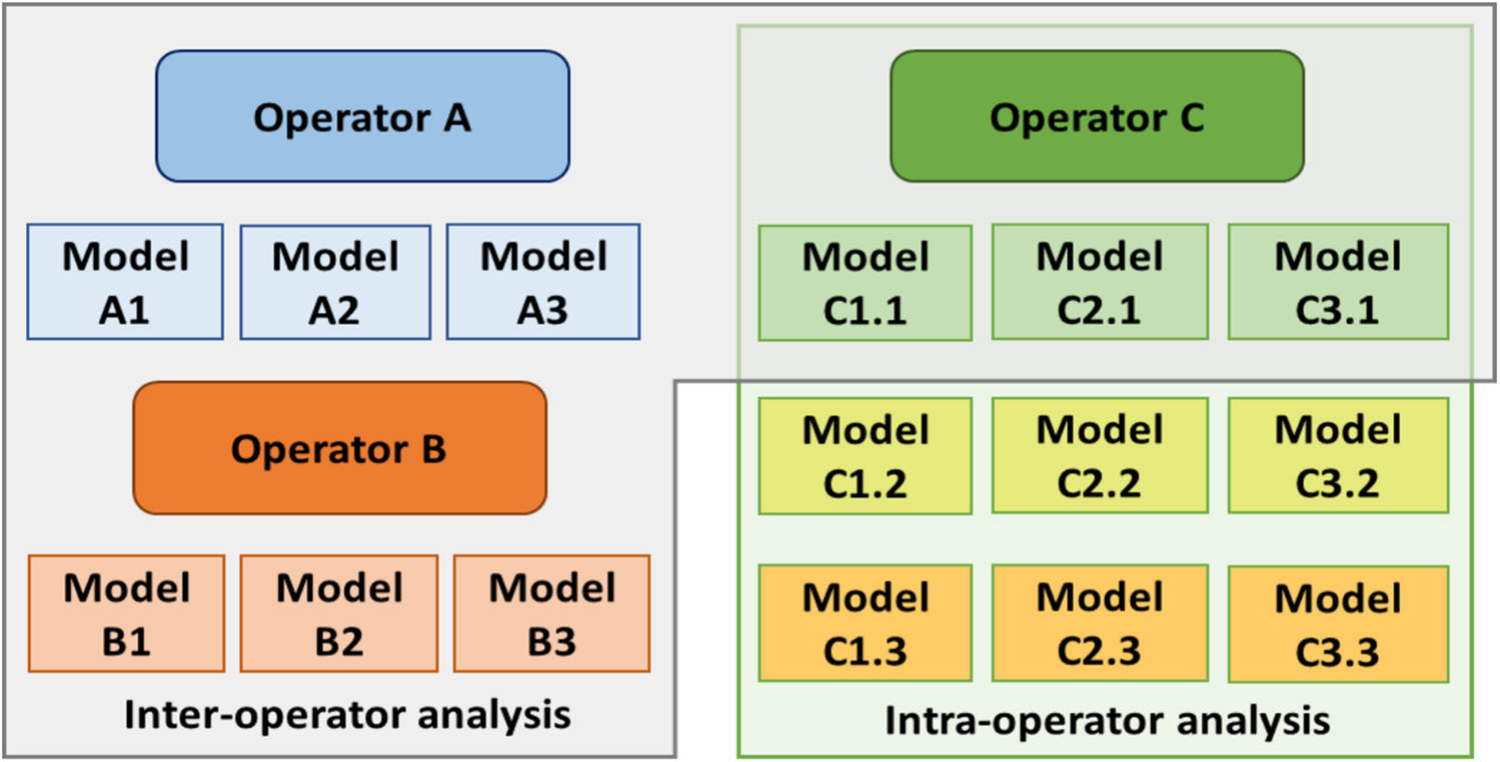
Outline of the repeatability study.

The above modelling procedure entailed two operator-dependent steps: the identification of muscles origins, insertions and *via* points; and the selection of the joints’ surface for morphological fitting (and consequent definition of joint frames, including their centre and axes orientation). Three operators were hence enrolled to assess both inter- and intra-operator variability of the procedure and their effects on the model output (Fig. 2). They produced three MSK models each using data from three randomly selected subjects (two females, one male, 13.7 ± 1.2 years, 1.63 ± 0.10 m, 68.5 ± 5.3 kg). One of the operators was also asked to repeat the modelling three times for each subject. Intra- and inter-operator variability of joint angles, joint moments and JCFs were quantified by standard deviation (SD) and range between repetitions over the entire gait cycle.

### Imaging Evaluation Assessment

An MRI-based assessment of joint involvement was performed for the hip, knee, ankle, and mid-foot joints. For each joint the MRI inflammation score was assigned on the short tau inversion recovery (STIR) sequence using a 0–3 scale based on the amount of joint effusion (0 = no inflammation; 1 = mild/moderate inflammation; 2 = severe inflammation). After training and calibration sessions, the MRIs were read by a paediatric radiologist and a paediatric rheumatologist with more than 10 years expertise in musculoskeletal MRI. The readers were blind to the clinical status of the patient. Any disagreement was resolved by consensus.^9^ This evaluation highlighted an active disease in 21 out of the 36 investigated limbs (Table 1). A total MRI score (*MRI*_Index_) was then calculated by adding the values of both lower limb joints and was used to divide the patients into two groups: impaired (*IM*, *n* = 13) and non-impaired (*NI*, *n* = 5). The *NI* group was then used as a control group.

### Statistical Analysis

A 1D non-parametric *t* test was used to compare joint angles, moments (normalised to body mass times height^21^), powers and contact forces (normalised to body weight, *BW*), estimated with the MSK simulations in the *IM* and *NI* by means Statistical Parametric Mapping (SPM) in MATLAB, using the SPM1D package.^25^

Each patient’s walking biomechanics was characterised using peaks of the hip (*F*_H1_, *F*_H2_), knee (*F*_K1_, *F*_K2_) and ankle (*F*_A_) JCFs; area under the hip (*A*_FH_), knee (*A*_FK_) and ankle (*A*_FA_) JCF curves; peak of ankle power (*P*_A_) and area under the hip (*A*_PH_), knee (*A*_PK_) and ankle (*A*_PA_) JP curves. For the *IM* group, the link between joint impairment and the biomechanical alterations was investigated analysing the correlation between the *MRI*_Index_ and the JCFs using the cumulative parameter including both limbs’ joints: *JCF*_Index_=* F*_H1_ + *F*_H2_ + *F*_K1_ + *F*_K2_ + *F*_A_. Observed correlations were classified as weak (0.3 < *ρ* ≤ 0.5), moderate (0.5 < *ρ* ≤ 0.7) or strong (*ρ* > 0.7), based on the Spearman’s Rho non-parametric test.

The *IM* group was sub-divided into patients with mono-lateral impairment (*MI*, *n* = 5), and patients with bilateral impairment (*BI*, *n* = 8) to investigate differences between relevant gait patterns. Dunn’s non-parametric multiple *t* test (critical *Q* value at 2.388) was used to highlight differences in the biomechanical parameters among *MI*, *BI* and *NI* using multiple, stepdown comparisons.^4^ Robust *z* score, based on outlier-insensitive median and median absolute deviation,^30^ was used to normalise the parameters and quantify the deviation of the *MI* and *BI* groups from the *NI* group, intended as a control.

Finally, the presence of contralateral compensatory strategies was quantified testing the correlation (Spearman’s Rho non-parametric test) between the *MRI*_Index_ of one limb and biomechanical parameters measured for the same limb and for the opposite limb. Significance was set to α = 0.05 for all the statistical tests.

## Results

**Figure 3:**
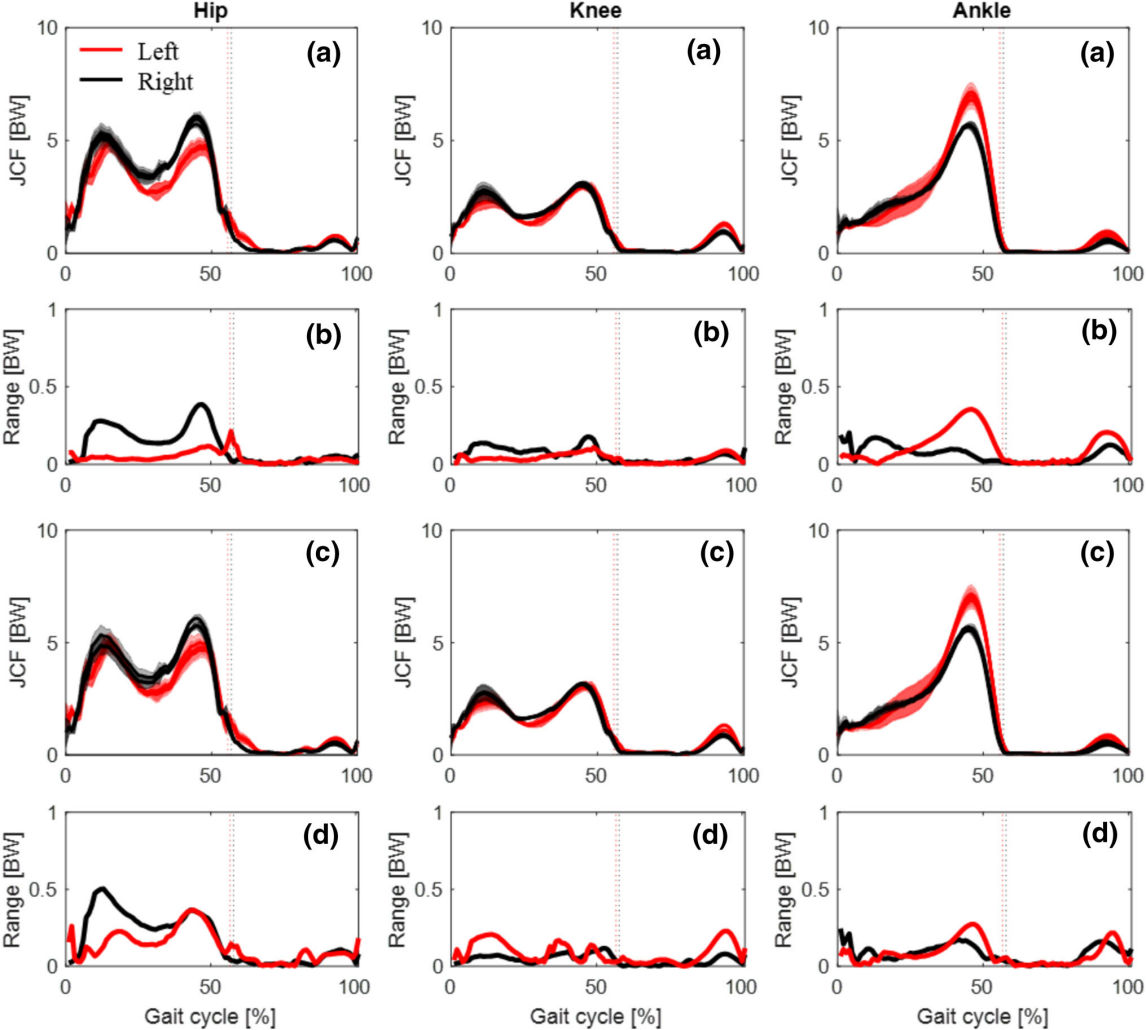
Repeatability of the model output: example of mean and SD (shadow) over three walking trials of hip, knee and ankle JCFs for one model (left and right side in red and black, respectively) built by the same operator three times (a) and three different operators (c). Ranges of variation of JCFs for (b) intra-operator and (d) inter-operator analysis

The three operators detected the muscle origins and insertions with an intra- and inter-operator variability of 1.2 ± 0.6 mm and 2.2 ± 1.0 mm, respectively. Intra- and inter-operator SD in the identification of the joint centres and axes orientation from morphological fitting was below 3 mm and 3°, respectively (Table 2), except for one model where intra- and inter-operator SD of the ankle axes orientation reached 5.2° and 8.3°. The propagation of these uncertainties to the models’ output, led to a maximum SD of joint angles and moments which was always below 3.0° and 0.03 N kg^−1^, respectively, for the intra-operator analysis and below 3.6° and 0.05 N kg^−1^, respectively, for the inter-operator analysis. The average percentage of SD with respect to the range of motion (ROM) was always below 10% except for the inter-operator SD of two models’ subtalar angles (Table 3). Intra- (Fig. 3a) and inter- (Fig. 3c) operator variations of the JCFs and their variations between-repetition (Figs. 3b and 3d) were all below 0.3 BW and 0.5 BW (equivalent to less than 10% of peak value).

**Table 2:** Repeatability of operator dependent input.

	Joint centre (mm)		Axes orientation (°)	
	Intra	Inter	Intra	Inter
Hip	0.2 ± 0.1	0.2 ± 0.1	1.6 ± 0.9	0.9 ± 0.2
Knee	1.3 ± 1.6	2.0 ± 0.8	1.7 ± 1.1	1.6 ± 0.5
Ankle	0.5 ± 0.1	1.0 ± 0.6	4.0 ± 1.8	3.9 ± 3.8
Subtalar	0.8 ± 0.2	1.5 ± 0.7	1.0 ± 0.2	1.0 ± 0.3

Mean ± SD (across the three models) of the intra- and inter-operator SD of joint centre and axes orientation (defined as the average SD over the three joint axes) for the lower limb joints

**Table 3:** Repeatability of model output.

	Hip flex/ext	Hip ab/ad	Hip int/ext	Knee flex/ext	Ankle PF/DF	Subtalar inv/ev
Joint angles (% ROM)						
M1						
Intra	0.6 ± 0.3	1.5 ± 0.4	3.8 ± 0.7	0.6 ± 0.4	7 ± 2.3	9.5 ± 3.2
Inter	0.4 ± 0.3	2.7 ± 1.5	5.8 ± 2.1	0.5 ± 0.2	7.8 ± 1.3	12.2 ± 2
M2						
Intra	1.2 ± 1.2	3.6 ± 1.8	5.2 ± 4.6	1.0 ± 0.6	9.6 ± 6.3	5.6 ± 4.9
Inter	2.4 ± 0.3	7.6 ± 1.4	6.1 ± 2.5	2.6 ± 0.5	4.4 ± 0.9	16.8 ± 5.3
M3						
Intra	0.4 ± 0.1	2.0 ± 1.0	2.9 ± 0.2	0.4 ± 0.1	1.7 ± 0.7	4.0 ± 0.0
Inter	3.7 ± 3.7	2.7 ± 1.5	9.4 ± 3.6	1.7 ± 0.7	5.6 ± 3.2	4.2 ± 1.6
Joint moments (% PP)						
M1						
Intra	0.8 ± 0.1	0.5 ± 0.0	1.0 ± 0.6	0.7 ± 0.6	0.3 ± 0.1	1.6 ± 0.4
Inter	0.8 ± 0.1	0.5 ± 0.2	1.5 ± 0.1	0.7 ± 0.4	0.3 ± 0.0	2.9 ± 1.0
M2						
Intra	1.0 ± 0.5	0.9 ± 0.5	1.5 ± 0.5	1.3 ± 0.3	0.3 ± 0.0	3.0 ± 0.7
Inter	2.1 ± 0.8	1.0 ± 0.2	3.3 ± 0.2	2.9 ± 0.6	0.8 ± 0.0	7.5 ± 2.6
M3						
Intra	0.4 ± 0.0	0.6 ± 0.1	0.8 ± 0.3	0.9 ± 0.4	0.2 ± 0.0	1.5 ± 0.3
Inter	0.9 ± 0.6	0.8 ± 0.0	8.4 ± 8.5	1.9 ± 0.9	0.5 ± 0.5	3.6 ± 2.8

Mean ± SD percentage of joint range of motion (ROM) and peak-to-peak moment (PP) for the intra- and inter-operator SD over the gait cycle for the three models (M1–3)

**Figure 4:**
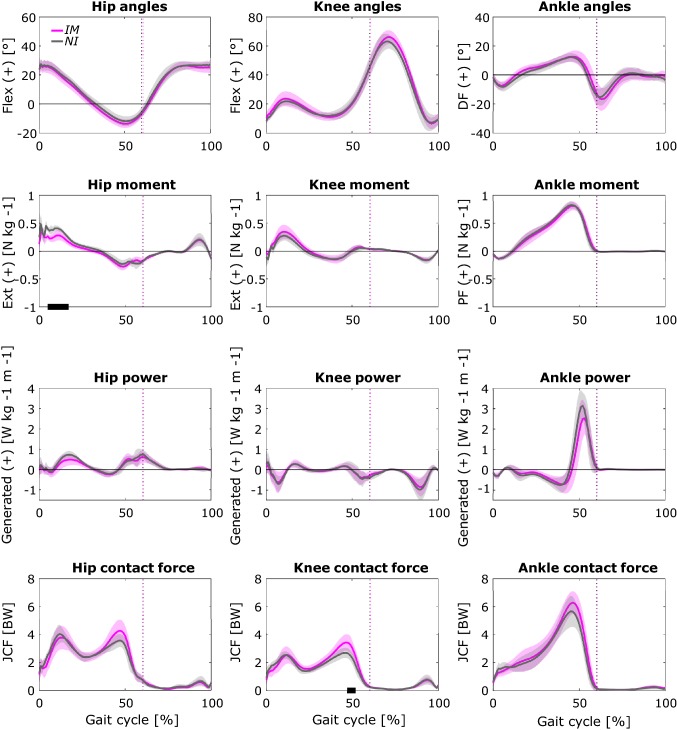
Comparison (non-parametric 1D *t* test in SPM) between joint angles, moments, powers (Abs = absorbed, Gen = generated) and contact forces of the *IM* (fuchsia) and *NI* (grey) groups over the gait cycle. Vertical dotted lines represent the instant in which toe off occurs and black bars identify the regions of the gait cycle where statistical significance was meet (*p* < 0.05).

The 1D *t* test between the *IM* and *NI* groups (Fig. 4) showed a significant difference only in the early stance phase of the hip moment, where the *IM* average joint flexion moment was up to 0.4 N m kg^−1^ smaller than *NI*, and in the second peak of the knee contact force, where the *IM* average JCF was up to 0.8 BW higher than the *NI*. All the remaining time-dependent comparisons were not significant.

**Figure 5:**
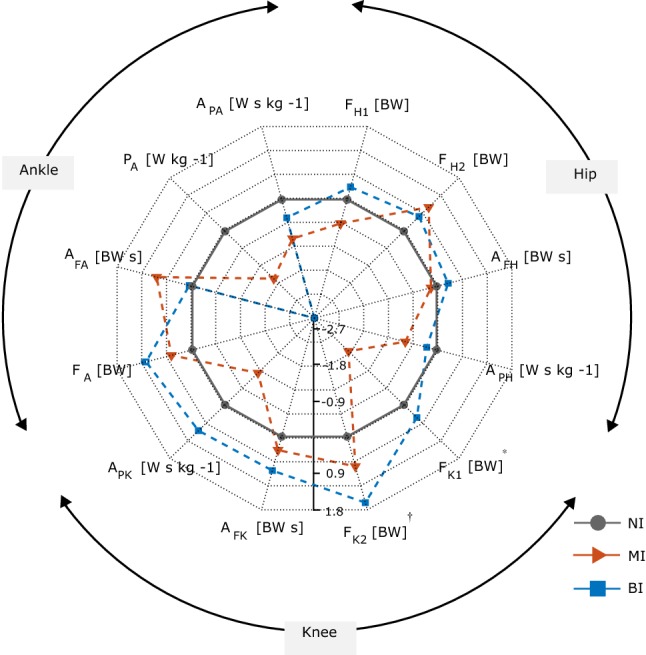
Radar plot visualisation of the JCF and JP parameters normalised using robust *z* score. * = *BI* group significantly different from *MI*; ^†^ = *BI* group significantly different from *NI*.

Table 4 shows the values obtained for the biomechanical parameters in the three groups. A meaningful statistical analysis was hindered by the low sample size, but the values did not seem to suggest a clear trend in the differences between the two limbs within the groups. Relevant values were then grouped to calculate the normalised *z* score used to build the radar plots in Fig. 5, which summarises the deviation of *BI* and *MI* groups from the biomechanical pattern shown by the *NI* groups. Visual analysis of the graphs suggests an overall tendency of *BI* to excessively load the knee when compared to the other two groups. The largest differences were observed for the *P*_A_ (*z* = − 3.0 and *z* = − 1.6 for *BI* and *MI*, respectively), *F*_K1_ (*z* = − 1.9 for *MI*), *F*_K2_ (*z* = 1.7 for *BI*), *A*_PK_ (*z* = − 1.1 for *MI*), *F*_A_ (*z* = 1.1 for *BI*). Peculiarly, *F*_K1_ and *A*_PK_ showed a discordant deviation, with positive *z* score for the *BI* and negative *z* score for the *MI*. Dunn’s test (*Q*_critic_ = 2.388) highlighted a significantly higher *F*_K1_ (*Q* = 2.8468) in the *BI* group compared to the *MI* (with 0.6 BW average difference) and *F*_K2_ (*Q* = 4.0224), in the *BI* group compared to the *NI* (with 1 BW average difference).

**Table 4:** Inter-group analysis.

		BI (n = 16)		MI (n = 10)		NI (n = 10)
		Most affected limb	Less affected limb	Affected limb	Non-affected limb	
		${\bar{\text{X}}}$ (range)	${\bar{\text{X}}}$ (range)	${\bar{\text{X}}}$ (range)	${\bar{\text{X}}}$ (range)	${\bar{\text{X}}}$ (range)
Hip	F_H1_ (BW)	3.9 (2.6/5.8)	4.2 (3.4/5.5)	3.7 (3.2/4.2)	3.3 (2.9/4.1)	3.9 (3.1/5.2)
	F_H2_ (BW)	4.1 (3.8/6)	4.7 (3.6/6.1)	3.8 (3.4/3.9)	4 (3.7/5.6)	3.8 (2.7/4.3)
	A_FH_ (BW s)	1.4 (1.6/2.3)	1.9 (1.7/2.4)	1.7 (1.5/1.7)	1.9 (1.6/2.1)	1.8 (1.6/2.1)
	A_PH_ (W s kg^−1^)	0.3 (0.1/0.4)	0.3 (0.2/0.4)	0.2 (0.1/1.0)	0.2 (0.1/0.3)	0.3 (0.2/0.4)
Knee	F_K1_ (BW)	2.6 (2.1/4.5)	2.7 (2.2/3.6)	2.0 (1.9/3.5)	2.1 (1.7/3)	2.5 (2.2/3.1)
	F_K2_ (BW)	3.7 (2.9/4.4)	4 (3/5.1)	2.8 (2.6/3.2)	3.4 (2.9/3.9)	2.7 (2.3/3.5)
	A_FK_ (BW s)	1.3 (1.1/1.5)	1.4 (1.2/1.6)	1.3 (1.1/1.6)	1.3 (1.1/1.7)	1.2 (1.1/1.5)
	A_PK_ (W s kg^−1^)	0.3 (0.1/0.5)	0.3 (0.1/0.4)	0.2 (0.1/0.4)	0.2 (0.2/0.3)	0.3 (0.2/0.5)
Ankle	F_A_ (BW)	6.6 (5.4/8.1)	6.4 (5.3/7.7)	6.0 (5.2/7.2)	6.3 (5.5/7.4)	5.7 (4.3/7.7)
	A_FA_ (BW s)	1.9 (1.5/2.2)	1.8 (1.6/2.2)	1.9 (1.7/2.6)	2.0 (1.7/2.1)	1.8 (1.4/2.1)
	P_A_ (W kg^−1^)	2.7 (2.4/4.8)	2.9 (2.2/4.3)	2.4 (1.9/3.3)	2.9 (2/3.4)	3.9 (2.2/4.7)
	A_PA_ (W s kg^−1^)	0.4 (0.3/0.5)	0.4 (0.3/0.5)	0.3 (0.3/0.9)	0.4 (0.3/0.5)	0.4 (0.2/0.5)

Medians (${\bar{\text{X}}}$) and ranges of the JCF and JP parameters for the three groups with n representing the number of limbs in each group

**Figure 6:**
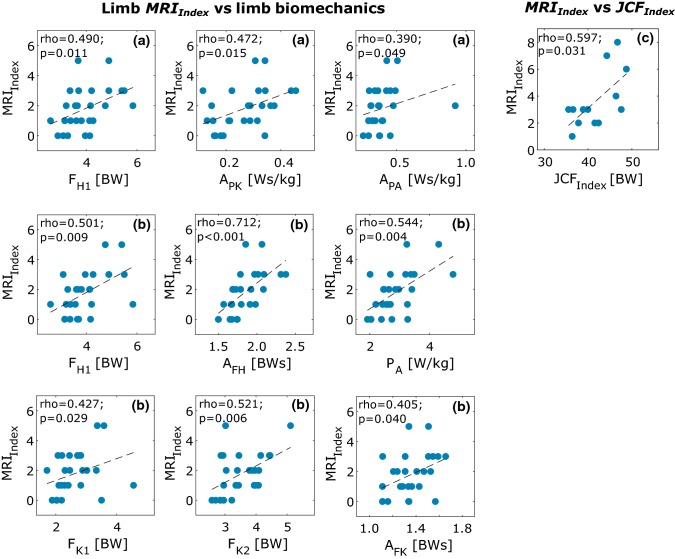
Spearman’s *ρ* non-parametric correlation between: (a) the *MRI*_Index_ of a single limb and the biomechanical parameters of the same limb, (b) the *MRI*_Index_ of a single limb and the biomechanical parameters of the contralateral limb, (c) the total *MRI*_Index_ and the sum of JCF peaks (*JCF*_Index_) of the two limbs. Dashed black lines represent linear regression fitting; *ρ* and *p* are the correlation coefficient and statistical significance, respectively.

A moderate correlation (*ρ* = 0.597, *p* = 0.031) was observed between the *MRI*_Index_ and *JCF*_Index_ (Fig. 6). When observing the link between *MRI*_Index_ of a single limb and the biomechanical parameters, a significant weak correlation was observed only for the *F*_H1_ (*ρ* = 0.490, *p* = 0.011), *A*_PK_ (*ρ* = 0.472, *p* = 0.015) and *A*_PA_ (*ρ* = 0.390, *p* = 0.049). When analysing the compensatory mechanisms involving the contralateral limb, significant weak to strong correlations were found for *F*_H1_ (*ρ* = 0.501, *p* = 0.009), *A*_FH_ (*ρ* = 0.712, p < 0.001), *P*_A_ (*ρ* = 0.544, *p* = 0.004), *F*_K1_ (*ρ* = 0.427, *p* = 0.029), *F*_K2_ (*ρ* = 0.521, *p* = 0.006), and *A*_FK_(*ρ* = 0.405, *p* = 0.040).

## Discussion

In this study, we proposed an MRI-based MSK model of the lower limb to investigate the relationship between joint impairment and joint loading during gait in a cohort of children with JIA, which was characterised by a variety of disease manifestations and consequent gait alterations. The reported results discouraged any hypothesis of a unique predictable cause–effect relationship, which suggests that adding a dynamic functional gait assessment to the image-based patient evaluation might help to better identify joints at risk of critical compensatory overloading and hence better inform personalised treatment. Furthermore, it clearly emerged that patient-specific models do have an ability to combine multiple data into coherent, physics-based predictions that appear to be strongly discriminative even in a dramatically heterogeneous population like the one investigated here. Thus, these methods should be pursued to clinically investigate the complex compensatory strategies that JIA flares produce and the effect that such strategies may have on the response to first line treatments.

The model adopted in this study presented some limitations. Firstly, the joints were schematised as ideal joints. This simplification is commonly accepted for the hip, being well described by the ball-and-socket coupling but represents a limitation in the understanding of knee and ankle motion.^34^,^44^ A second limitation was the estimation of musculotendon parameters. They were linearly scaled to lower-limb mass from a generic model available in the literature 11 where these parameters were specified for only a single nominal subject. Experimental data suggest that musculotendon parameters are highly variable between subjects, especially when anthropometrical differences are considerable (i.e., children vs. adults), therefore linear scaling of these quantities might not be appropriate for a juvenile population. On the other hand, a direct and non-invasive measure of these parameters is not possible *in vivo*. Future study will aim at improving this aspect, implementing methods to extract more information from MRI (such as moment arm, individual muscle volume and cross-sectional area). Modenese *et al*.^20^ showed that the choice of the scaling method does not influence the resulting JCFs if the FLV relationship is taken into account. Here, on the contrary, contraction dynamics was neglected, potentially causing the overestimate of the second knee contact force peak.^20^ However, the consistency of this choice throughout all the simulations did not affect the comparison between impaired and non-impaired subjects’ JCFs. Finally, we applied the Static Optimisation technique to estimate muscle forces assuming an optimal force distribution strategy. This might not be the case in a pathological population, where suboptimal mechanisms can be adopted aiming at reducing joint loading.^12^

Despite the above limitations, the proposed approach led to satisfactory intra- and inter-operator repeatability of the estimated output in the context of the investigated application. The variability observed in the input did not substantially affect the output of the simulations, with limited variations observed for all joint kinematics and kinetics and for the joint contact forces. The combined effect of mis-locating joint centre and axes and misidentifying muscle points led to an overall uncertainty of 0.5 BW, which is lower than 10% of the estimated peak values. The operator-related uncertainty found in the repeatability study was considered reasonable to safely apply the modelling protocol in a clinical scenario to estimate joint angles, moments, powers and contact forces, and to investigate the link between joint impairment and alteration of the relevant biomechanical parameters in JIA. Lower repeatability was observed for the movements out of the sagittal plane, for this reason, only flexion/extension movements were investigated in this study. This choice is certainly a limitation, but it is in line with previous gait analysis studies on JIA children.^3^,^15^,^19^,^22^

The uncertainty in the identification of the joint centre and axes was similar to what has been reported in the literature^18^ for the knee and ankle joints of healthy adults (up to 6.4 mm and 4.5° and up to 4.6 mm and 4°, respectively), leading to JCF variations of up to 9% of peak values. Previous studies demonstrated that the repeatability of JCF estimates is highly dependent on the definition of muscle geometries. Navacchia *et al*.^23^ showed that muscle path uncertainty can have an average 10% effect on the predicted JCFs. In a previous study investigating a juvenile ankle model,^14^ our group reported up to 20% of peak ankle JCF variability due to intra- and inter-operator uncertainties in muscle point identification equal to 1.7 ± 1.9 and 3.0 ± 2.5 mm, respectively, with maximum values up to 14.3 mm for single points. The intra- and inter-operator variability of muscle points in the present study was reduced to 1.2 ± 0.6 and 2.2 ± 1.0 mm, respectively, with maximum values of 5 mm. This progress was the result of an improved identification of the set of bony landmarks used for the supervised registration of muscle points.^20^ Nonetheless, since muscle paths are a well-known critical factor in the estimate of moment arms, muscle forces, and JCFs,^33^ future investigations should focus on further automating and improving this step.

The modest propagation of the input uncertainty on the models’ predictions made these patient-specific models highly discriminative; we were able to highlight significant differences between individual patients, and between limbs in the same patients. However, the cohort of children enrolled in this study was characterised by a high clinical heterogeneity with different JIA subtypes and severity. Five children, despite a history of JIA, did not present active disease at the time of the visit, and were therefore classified as not impaired. Eight patients presented bilateral impairment and five mono-lateral impairment, mostly affecting the knee and ankle joints. This heterogeneity clearly affected the results of the group analysis of disease-related gait pattern, due to a large variability in the average joint angle, moment, and power curves. Consequently, no specific pathology-related pattern was detected in the *IM* group kinematics, contrary to what was reported in a previous study,^15^ where hyper-flexion of the hip and knee joints and reduced plantarflexion in the ankle were found to be a common trait in 36 patients with symmetrical polyarticular joint involvement. A possible explanation for this discrepancy can be found in the reduced numerosity (n = 5) of our non-impaired group, and in the fact that JIA-related joint inflammation had been reported for these children within the previous 12 months. As such, rather than fully representative of a healthy population, their gait biomechanics was that of a group of individuals capable of responding to the disease activity by leveraging on loading and walking strategies that enabled them to reduce joint inflammation and pain.

The limited number of participants and the variability of their clinical status drove the choice of using a cumulative impairment scoring index (accounting for all the lower limb joints), which prevented the investigation of individual contributions of each joint and the impact of different involvement levels to the overall functional alteration. Larger and more homogeneous datasets would be necessary to overcome this limitation. Nonetheless, the cumulative *JCF*_Index_ was found to be moderately correlated to disease activity level in the lower limbs, and when analysing the joints separately, a significantly higher (up to 15%) knee peak contact force was observed during push off in the *IM* group. This result was partially confirmed when investigating distinctive features of mono-lateral and bilateral impaired groups. In fact, a positive *z* score was observed for all the JCF parameters of the *BI*, resulting in the overloading of the joints, with particular significance for the knee (*F*_K1_ and *F*_K2_).

When the behaviour of the two limbs was investigated separately, the presence of compensatory loading strategies became evident, with an increased loading of the contralateral hip (higher *A*_FH_) in the most impaired patients. Additionally, overloading of the hip arose for both limbs in the first phase of the stance (higher *F*_H1_). An overall higher loading of the knee (*A*_FK_) was observed, especially during push-off phase (*F*_K2_), in the less affected limb as a possible strategy for protecting the painful joints. This excessive loading might be one of the causes for further development of the pathology. From this perspective, the knee joint loading might be the best variable to monitor in order to predict disease progression and guide treatment.

In conclusion, this paper presented for the first time the application of a juvenile subject-specific MSK modelling approach to the investigation of the link between joint impairment and joint loading during walking in children with JIA. The model ensures repeatable estimates of lower-limb biomechanical parameters and the results of its application encourage further development of this approach as a support of the current clinical practice for understanding and preventing functional alterations associated to excessive joint loadings. In this sense, only knee JCF resulted as a good candidate for predicting JIA activity and potential indicator of compensatory mechanism associated to mono-lateral involvement, but future longitudinal studies are needed to test this hypothesis.
